# Hand Transplantation Versus Hand Prosthetics: Pros and Cons

**DOI:** 10.1007/s40137-016-0128-3

**Published:** 2016-01-27

**Authors:** S. Salminger, A. D. Roche, A. Sturma, J. A. Mayer, O. C. Aszmann

**Affiliations:** Division of Plastic and Reconstructive Surgery, Department of Surgery, Medical University of Vienna, Waehringer Guertel 18-20, 1090 Vienna, Austria; Christian Doppler Laboratory for Restoration of Extremity Function, Medical University of Vienna, Vienna, Austria; Department of Plastic Surgery, Southmead Hospital, North Bristol NHS Trust, Bristol, UK; Master Degree Program Health Assisting Engineering, University of Applied Sciences FH Campus Wien, Vienna, Austria

**Keywords:** Hand amputation, Hand transplantation, Hand prosthesis, Upper extremity, Reconstruction

## Abstract

Composite tissue transplantation and new developments in the field of prosthetics have opened new frontiers in the restoration of function among upper limb amputees. It is now possible to restore hand function in affected patients; however, the indications, advantages, and limitations for either hand transplantation or prosthetic fitting must be carefully considered depending on the level and extent of the limb loss. Hand transplantation allows comprehensive hand function to be restored, yet composite tissue transplantation comes with disadvantages, making this method a controversial topic in the hand surgical community. Alternatively, prosthetic limb replacement represents the standard of care for upper limb amputees, but results in the known limitations of function, sensation, and usage. The indication for hand transplantation or prosthetic fitting strongly depends on the level of amputation, as well as on the extent (unilateral/bilateral) of the amputation. In this review, we discuss the advantages and disadvantages of hand transplantation and prosthetic replacement for upper limb amputees in general, as well as in regard to the different levels of amputation.

## Introduction

Young people are particularly at risk of upper limb amputations, as trauma is the leading cause [1, 2]. This is in contrast to lower limb amputees, which mainly occur in elderly patients with end-stage vascular diseases or diabetes [3]. Hands are needed for almost every activity of daily life. In addition, hands are an essential part of our appearance, are important for our physical and psychological development, and play a significant role in determining our professional career [4–6]. These facts highlight the importance and necessity of reliable replacement of upper limb function. Attempts to replace this highly sophisticated organ have been developed over the past 70 years in both the fields of surgery and prosthetic reconstruction [7••, 8].

Composite tissue transplantation and improvements in the field of prosthetics have opened new frontiers in restoring hand function. The first documented hand transplantation was performed in Ecuador in 1964 [9]. Due to insufficient immunosuppressive treatment, the hand had to be removed 2 weeks later [10]. A group in France then performed the first successful human hand transplant in 1998 [11]. Since then, 107 upper extremity transplantations in 72 patients have been performed in 26 centers worldwide [12••].

Historically, the first electronically-driven hand prostheses were developed towards the end of World War Two [13]. Cosmetic features, weight savings, battery life, and components have improved over time, and prosthetic fittings with myoelectric devices have been established as the standard of care in upper limb amputees [14]. These myoelectric systems are controlled by a minimum of two individual muscle groups at the remnant limb of the amputee [13]. Prosthetic fitting can take place soon after the initial injury with short hospitalization and rehabilitation [2].

Hand transplantation and prosthetic fitting both have their advantages and limitations. Unlike solid organ transplantation, limb transplantation involves the risk of a shortened life expectancy but may improve its quality. Therefore, the risk–benefit ratio becomes far more delicate, subjective, and hence controversial. In this review, we will discuss the advantages and disadvantages of hand transplantation and prosthetics for upper limb amputees in general, as well in regard to the different levels of amputation.

## Hand Transplantation: Advantages and Limitations

Hand transplantation perfectly fulfills Sir Harold Gillies’ concept of “replacing like with like,” while avoiding donor-site morbidity [10]. Successful hand transplantation replaces the lost body part with a limb that is silent, worn constantly, never exhausted, aesthetically pleasing, warm to touch and hold, and with the self-repairing qualities of the biologic tissue. Hand transplantation does not only restore motor function but also enables a sense of touch, bodily integrity, ownership, and wholesomeness [15]. Although functional outcomes are not reported as consistently, different groups show excellent and long-lasting results [16•, 17].

Apart from long-lasting rehabilitation and recurrent inpatient treatment, the need for immunosuppression is the greatest risk of composite tissue transplantation. The possible side effects of immunosuppressive drugs are well known and not to be ignored, with every patient experiencing at least one acute episode of rejection [18, 19]. The number of such episodes may be important, as rejection has a negative influence on sensory and motor recovery [20]. Although some authors state that selected patients for transplantation are otherwise healthy and most probably do not have comorbidities that impact on the side effects [10, 21, 22], considering all possible complications, lifelong immunosuppression is not to be underestimated. As chronic hypertension increases the risks of vascular infarction, long-lasting immunosuppression increases the risk of infection, neoplasia, metabolic disorders or organ failure [23, 24]. Furthermore, the need for immunosuppression requires the taking of a considerable amount of drugs according to a strict time schedule. Blood samples need to be taken frequently to monitor side effects such as nephrotoxicity, and other investigations are necessary for follow-up. Despite strict transplant care, the risk of acute rejection remains high [10]. Thus, in the latest review of the worldwide experience with hand transplantation, 24 graft losses were reported (22.4 % loss rate for all limbs) due to patient death, acute or chronic limb loss [12••].

As can be seen in the first case from France, non-compliance with immunosuppression and physical rehabilitation leads to poor functional outcome. In that case, Merle described hand function as “effectively a paperweight.” [25] Considering the re-amputation of the French patient, Cooney et al. asked a decisive question: “How good is the achievable ‘quality of life’ with a new limb when daily medications are required to maintain its viability?” [26] However, if patient selection and postoperative treatment are adequate, hand transplantation can achieve excellent hand function and a tremendous improvement in the patient’s quality of life.

## Hand Prosthetics: Advantages and Limitations

Prosthetic replacement in upper limb amputees has for many years been considered as the standard of care [13, 14]. Yet, prosthesis use is notoriously challenging for activities like carrying out body hygiene or grooming. Therefore, different prosthetic attachments can be custom-fitted for different tasks in daily life. However, the abandonment of expensive prosthetic devices represents an economic problem and a burden for all different professions involved in the prosthetic fitting of upper limb amputees. A literature review from 2007 observed an average rejection rate of all prostheses (cosmetic, body-powered, myoelectric) in 1 out of 5 individuals with upper limb deficiency over the last 25 years [27].

A myoelectric prosthesis is able to replace sufficient motor function to aid in daily life activities; however, the lack of sensory information leads to difficulties in performing precise motor commands as visual control is mandatory [7••]. The missing sensory feedback represents a major burden especially in bilateral amputees.

However, prosthetic fitting can take place early after the injury, and controlling the prosthesis especially for below-elbow amputees is mostly intuitive and easy to learn in an adequate rehabilitation setting. Furthermore, no additional surgery or life-long medication is needed to fit an upper limb amputee with a prosthetic device, and patients can return to near normal life reasonably quickly [14].

## Indications

In composite tissue transplantation, which represents a surgically and immunologically invasive procedure with the necessity of life-long medication, patient selection is crucial. According to the principle of *Primum non nocere*, a surgical intervention is indicated if the patient’s life time can be prolonged, their condition be improved and additional risks are acceptable and outweighed [28]. Following the first successful transplantations, this procedure was considered as a new avenue in upper extremity reconstruction; nevertheless, not all hand surgeons supported the concept of reconstructive transplantation [6]. A survey by Mathes et al. revealed that bilateral below-elbow amputation was the most accepted indication for hand transplantation by hand surgeons of the American society (78 %), whereas only 32 % also supported hand transplantation in patients after unilateral amputation of the dominant hand [29]. A majority (69 %) of the respondents in this survey assessed hand transplantation as a high-risk endeavor, and were either against hand transplantation (45 %) or undecided (31 %) [29]. However, this survey is from 2009 and attitudes towards hand transplantation might have changed.

After amputation of the dominant hand, the healthy non-dominant hand will most probably become dominant [10]. Even in bilateral transplant patients, changes of the dominant hand and dexterity after transplantation have been reported [30]. In unilateral amputees, the reconstructed hand, either a transplanted or a prosthetic one, will be a helping hand [31]. Probably more important than dominant or non-dominant is the fact that patients who lose their right hand feel uneasy and intimidated because they cannot shake hands in an ordinary manner. In unilateral transplantations, the difference between both hands in size, skin texture and color or hair growth will always be noticeable [31], although the aesthetic appearance with transplantation will always be superior to a prosthetic hand. Nevertheless, individuals’ views of risk can differ greatly. A survey of amputee patients, organ transplant recipients and healthy subjects showed that hand/arm amputees did not see a great benefit in a single hand transplant [32]. The amputee patients were significantly less willing to accept the risks of a single hand transplant than the group of organ transplant recipients and also less willing to accept a single hand transplant compared to the healthy volunteers [32]. The risk acceptance for a bilateral hand transplantation was nearly twice as high as for unilateral transplantation, and the organ-transplanted group were willing to accept nearly the same amount of risk for bilateral hand or kidney transplantation [32]. These results suggest that amputee patients are coping effectively with one functioning hand and their prostheses, and therefore the risk acceptance in regard to immunosuppression is low. However, in unilateral amputees, psychological impairment is claimed as one of the major indications for hand transplantation [18, 33]. Additionally, a sensate stump in distal unilateral amputees is quite functional, and these patients can usually perform up to 90 % of the activities of daily living together with the sound arm [34, 35].

**Fig. 1:**
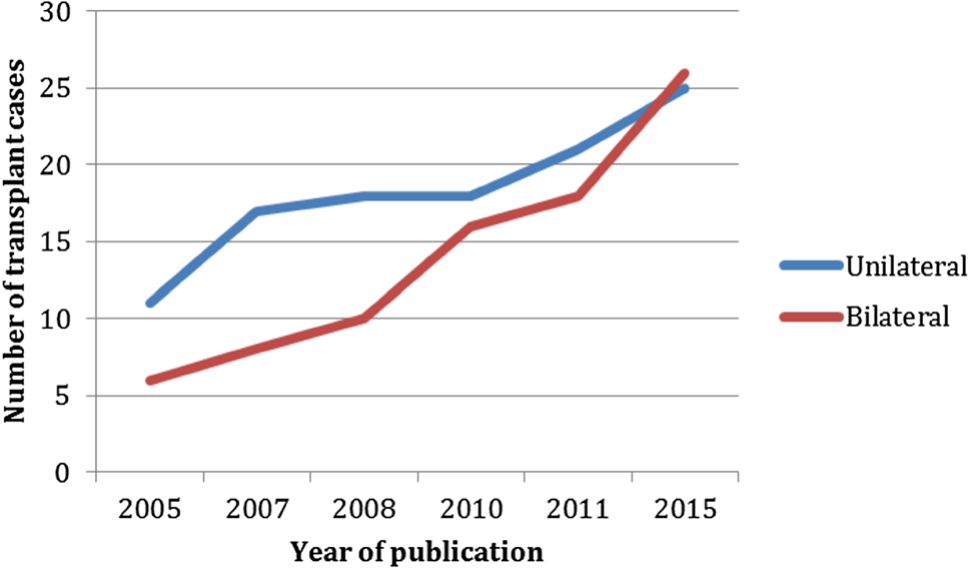
Relationship between unilateral and bilateral cases from the total number of hand transplantations performed

According to the latest report of the International Registry on Hand and Composite Tissue allotransplantation from 2011, 44 % of the transplanted patients were performed on bilateral amputees [19]. However, in a recent review covering the worldwide experience of upper extremity transplantation from Shores et al., all known cases were summarized, showing a paradigm shift within the last years [12••]. As can be seen in Fig. 1, unilateral hand transplantations were more common in the initial years, before bilateral transplantation became the main indication from 2008 onwards.

One of the major causes of upper limb amputations are explosion or burn injuries, also leading to visual impairments or blindness in some patients [36]. Performing hand transplantation in a blind amputee may provide him with both motor and sensory function, which cannot be offered by the current generation of prosthetic devices [37]. Nevertheless, the sensory feedback of a transplanted hand will not be comparable to the sensory capacity of the sound skin at the stump region. Therefore, hand transplantation in blind upper limb amputees is controversial [36–38].

Replantations have shown that the functional outcome is also dependent on the level of amputation: the higher the amputation, the less successful the outcome [39]. Thus, transplantations have been favored at the distal transradial or even wrist level [38]. The ideal patient for hand transplantation would be a bilateral distal transradial amputee suffering a sharp traumatic injury who is already under immunosuppression because of a life-saving procedure [40].

The motives driving patients to have hand transplantation are distinct. In general, unilateral amputees primarily report difficulties with coping and psychological issues, whereas patients with bilateral amputation especially suffer from functional impairment and loss of quality of life [18]. However, an overwhelming majority (71 %) of surgeons in the survey of Mathes et al. believe that hand transplantation does produce a benefit when performed on a properly selected patient [29]. Although requested by experts of this field, surprisingly, a comparison of hand function of transplanted hands with up-to-date prosthetic hands at a similar amputation level has not yet been performed [41]. The largest trial comparing hand function of prosthesis and replants was performed by Graham et al. [42]. Nevertheless, they include different amputation levels and different prosthetic devices.

## Rehabilitation

As Ninkovic et al. states, the clinical outcome after hand transplantation is strongly dependent on genetic matching, number of rejection episodes and the chosen immunosuppressive regimen, precise and accurate surgery and to a great extent on adequate rehabilitation [17]. The expense for rehabilitation in hand transplantation is tremendous. It needs a carefully selected patient who is willing to take the burden of several months of inpatient treatment and a life-long engagement for his hand. A single-center cohort of patients received an average inpatient treatment for 4.25 ± 5.02 months with 3-4 h of therapy for 7 days a week, and later an average outpatient treatment of 11.16 ± 9.31 months with 3–6 h for 5 days a week [17].

**Fig. 2:**
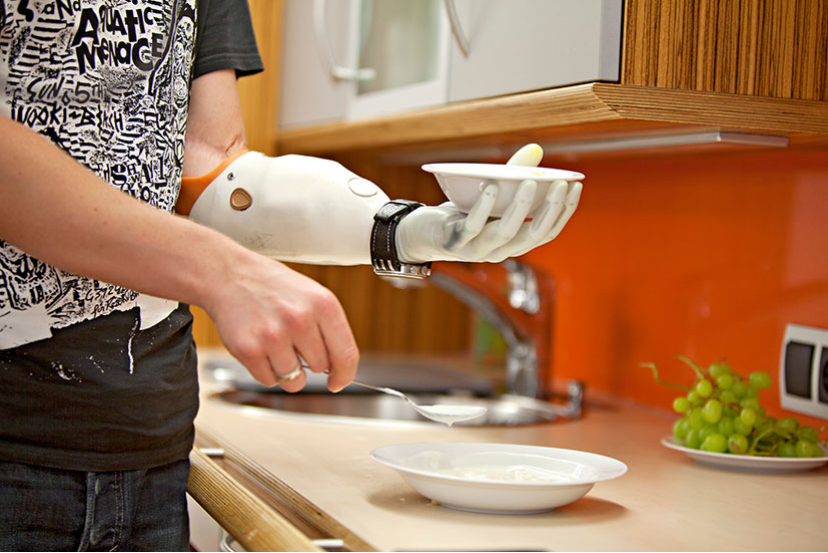
A prosthesis used in activities of daily living

Prosthetic fitting can be performed as early as 3 months post-amputation, after swelling of the stump has resolved and atrophy of the muscles is stable. This delivers a constant and to a great extent predictable outcome which can be further improved over time [14]. Prosthetic rehabilitation usually starts a few weeks after surgery and at best even before prosthetic fitting [43]. Since standard prostheses are controlled by two independent (mostly antagonistic) myoelectric signals, the voluntary contraction of the corresponding muscles is trained in therapy. Using electromyography (EMG) biofeedback devices, this can also be done without a prosthesis. Amputees usually learn how to control the two myoelectric signals within a few therapy sessions. As soon as the prosthesis is fitted, the focus is on using it in activities of daily living (Fig. 2). Different strategies on how to perform certain tasks are discussed and tested in therapy. This should enable the patient to use the prosthesis in a way that supports him/her in daily life. To our knowledge, there is no precise recommendation for the amount of therapy needed for a standard fitting. Nevertheless, in our experience, 10–20 h of therapy in total are usually enough. Only in cases with additional surgery to improve the man–machine interface (e.g., nerve or muscle transfers) may the rehabilitation time be prolonged [14, 44, 45].

## Costs

The costs of the reconstructive procedure play an important role for the public and private insurance providers. Other organ transplantations, such as liver and heart, which are even more expensive, are widely accepted because of the lack of alternatives in the treatment of life-threatening conditions. As stated earlier, hand or arm amputation is not a life-threatening condition and prosthetic devices do provide a reliable and less expensive alternative. Different financial factors have to be taken into account, including surgical costs, in- and outpatient treatment, occupational therapy, immunosuppressive drugs and the time out of employment [46]. Cost–utility analyses have been performed and have concluded that prosthetic fitting would be the preferred treatment for upper extremity amputees, both in uni- and bilateral cases [47]. Recently, the Swiss Health Care Association rejected hand transplantation as a treatment modality because of the fourfold costs of composite tissue transplantation compared to prosthetic fitting [48].

## Level-By-Level Analysis

### Below the Elbow

Below the elbow, including proximal and distal transradial amputations as well as the wrist level, represents the level of amputation with the best possible outcome either with prosthetic devices or hand transplantation [14, 17]. Thus, hand transplantation at the transradial or wrist level is the most common composite tissue transplantation [19]. However, the functional outcome of the hand in daily life is strongly dependent on the shoulder and elbow function of the patient. A good shoulder and elbow joint is essential to move the hand (transplanted or prosthetic device) in three-dimensional space.

In hand transplantation below the elbow, the patient’s own self-innervated extrinsic flexors and extensors are moving the transplant, and in some cases intrinsic muscle function will be regained after successful nerve regeneration. However, some hand function is present from the first day after surgery and therefore not dependent on the success of nerve regeneration.

Myoelectric prostheses for below-elbow amputees are controlled by two individual muscle groups of the remaining limb, using signals from one muscle group to open and another to close the hand, with some advanced devices allowing movements of the wrist or specific grip patterns [13]. Thus, controlling the prosthesis for a below-elbow amputee is mostly intuitive and easy to learn in an adequate rehabilitation setting.

### Above the Elbow

Above-elbow transplants are less frequent compared to the below-elbow amputation level, with six known transplantations worldwide [12••]. The distances for nerve regeneration and the number of muscles that need to be reinnervated are the major concerns in limb transplantation at the transhumeral level. As not only the intrinsics but also all finger/wrist flexor and extensor muscles are not working during the months of nerve regeneration, the course of rehabilitation is even longer and results in further obstacles for motor recovery [49]. Due to the distance from nerve coaptation to the distal hand, no recovery of the intrinsic muscles has been reported [50]. As Shores et al. state, the best to be expected is some forearm pronation/supination, wrist flexion/extension and enough extrinsic digit flexion/extension for the hand to function as a helping hand only [50].

Conventional myoelectric upper arm prostheses are controlled by surface electrodes that are sourced by two separately innervated muscle groups [14]. Since the prostheses usually have an elbow, wrist and hand joint, switching between these levels is necessary. This is achieved via co-contraction. Therefore, conventional control of above elbow prostheses is slow and unintuitive [14, 51]. To enhance prosthetic control at this amputation level, a new surgical option has been established over the last 15 years [52]. The multiplication of EMG sites with selective nerve transfers of the brachial plexus to the remaining stump muscles, known as the targeted muscle reinnervation (TMR) technique, has enabled prosthetic control in a way that was harmonious with the natural pattern of movement without the need to change between the different prosthetic joints [53]. The functional benefit of TMR in high-level amputees compared to conventional myoelectric or body-powered prostheses has been shown by various groups [14, 51–54].

### Shoulder

Limb transplantation is currently not proposed at this level of amputation, as the distance for nerve regeneration would be even longer than at the above-elbow level, as well as requiring an additional joint to be transplanted and with elbow function also dependent on nerve regeneration, compared to the above-elbow level [55]. Furthermore, skeletal attachment would be challenging.

This level of amputation represents a standard indication for TMR surgery, although prosthetic replacement with a functional myoelectric shoulder joint is only possible in the laboratory setting at this time [14]. These patients are fitted with a passive shoulder joint, and a myoelectric elbow, wrist and hand. The principle of the TMR surgery remains the same, with only the targeted muscles for nerve transfers changing. In regard to neuroma pain, these nerve transfers, as well as limb transplantation, take care of all amputation -neuromas, as they rewire each nerve to a useful distal target [14].

## Future Outlook

Hand transplantation as well as prosthetic devices will most definitely further improve within the next years. The successful induction of donor-specific tolerance would have great impact on the range of indication, as the risk of chronic rejection could be reduced or even eliminated, resulting in a safe transfer and most probably improved motor and sensory outcomes [31, 56]. On the other hand, new control algorithms such as pattern recognition will enhance the functionality and applicability of prosthetic devices. Recently, such an algorithm has become commercially available (Coapt) with future improvements on the horizon, and broad uptake can be expected in the near future [13, 27]. Ongoing research is focusing on providing the prosthesis user with tactile and proprioceptive feedback; however, to date, these systems are not available for clinical use [57]. Thus, future developments of prosthetic devices will have great impact on the indications for composite tissue transplantation [28].

## Conclusion

Hand transplantation poses a sophisticated opportunity to truly restore hand function in combination with sensation and self-perception, and therefore enhancing the quality of life in upper limb amputees [18]. Nevertheless, even 17 years after the first successful hand transplantation in human and an experience of over 100 transplanted limbs worldwide, composite tissue transplantation is still a controversial topic in the hand surgery community [46].

Although limb loss is not a life-threatening condition, losing one or even both hands leads to severe functional impairments as well as an immense psychosocial burden [31]. In bilateral limb loss, the benefits of motor and sensory restoration may outweigh the risks of immunosuppression, leading to superior outcomes compared to prosthetic fitting, especially in below-elbow amputees [58]. As unilateral amputees are able to compensate for the majority of the functional deficit using their remaining healthy hand and a prosthesis, the indication for hand transplantation should be focused on bilateral below-elbow limb loss [18, 58]. The ideal candidate would be a patient who is already under immunosuppression for a life-threatening condition and has lost both his hands [40]. This patient would benefit from all the advantages of hand transplantation without additional risks [5, 20]. Although some patients have reported that there were tasks like carrying heavy objects which were easier with the prosthesis, if hand transplantation is successful, overall functional outcome is superior to any current prosthetic device in below-elbow amputees [58, 59].
